# Synchronous and Metachronous Breast and Ovarian Cancer: Experience From Two Large Cancer Center

**DOI:** 10.3389/fonc.2020.608783

**Published:** 2020-12-14

**Authors:** Giulia Tasca, Maria Vittoria Dieci, Zora Baretta, Giovanni Faggioni, Marco Montagna, Maria Ornella Nicoletto, Fedro Alessandro Peccatori, Valentina Guarneri, Nicoletta Colombo

**Affiliations:** ^1^ Medical Oncology 2, Veneto Institute of Oncology IOV—IRCCS, Padova, Italy; ^2^ Department of Surgery, Oncology and Gastroenterology, University of Padova, Padova, Italy; ^3^ Oncology Unit, Hospital of Montecchio Maggiore, Montecchio Maggiore, Vicenza, Italy; ^4^ Immunology and Molecular Oncology Unit, Veneto Institute of Oncology IOV—IRCCS, Padova, Italy; ^5^ Istituto Europeo di Oncologia - IRCCS, Milano, Italy; ^6^ University of Milano-Bicocca, Milano, Italy

**Keywords:** metachronous cancer, synchronous cancer, breast cancer, ovarian cancer, doublet tumors, BRCA mutation

## Abstract

**Purpose:**

We aimed to evaluate the clinico-pathological characteristics and survival outcomes of patients with synchronous or metachronous breast cancer (BC) and ovarian cancer (OC).

**Materials and Methods:**

Patients with synchronous or metachronous BC and OC were retrospectively identified at two large cancer centers. Clinico-pathological characteristics, *BRCA1/2 status* and follow-up data were gathered. Patients were classified according to the first cancer diagnosis in the following groups: Breast Cancer *first*, Ovarian Cancer *first*, Synchronous Breast and Ovarian Cancer. Overall survival (OS) was calculated as the time interval between each cancer diagnosis to death or last follow-up.

**Results:**

Overall, 270 patients were included: n = 194 (72%) in *BC first* group, n = 51 (19%) in *OC first*, and n = 25 (9%) in *synchronous. BRCA status* was available for 182 (67.4%) patients and 112 (62%) harbored pathogenetic mutations. *BC first* group included more frequently patients with *BRCA* mutation, triple negative BC phenotype and more aggressive OC features. Median time between the two diagnosis was longer in *BC first* group *vs OC first* group (95 *vs* 68 months, p = 0.021). A total of 105 OS events occurred, mostly related to OC (70.5%). We observed no differences in terms of OS according to the first cancer diagnosis. Age >50 years and advanced OC stage were negative independent prognostic factors for OS from the first diagnosis.

**Conclusions:**

In this cohort of patients with BC and OC, survival was dominated by OC related mortality. These data may be useful to plan and carry out adequate and timely surveillance programs and preventive measures.

## Introduction

Breast cancer (BC) is the most frequent cancer and the leading cause of cancer death among females worldwide, with an estimated 1.6 million cases and 521,900 deaths in 2012 (1). Ovarian cancer (OC) is the 7th most frequent cancer diagnosis, with 238,700 new cases in 2012, and the 8th cause of cancer mortality, with 151,900 deaths (1).

When compared with the general population, cancer survivors have generally an increased risk of developing a second primary cancer at a different sites (2, 3). Register-based studies show that women with BC are at increased risk of developing OC and that long term OC survivors are at increased risk of developing BC cancer (4, 5).

In the early 1970s, Lynch provided the first evidence of an autosomal-dominant inherited trait predisposing women to both BC and OC (6, 7). In 1990 Mary-Claire King demonstrated that a single gene on chromosome 17, later known as BRCA1, was responsible for many breast and ovarian cancer (8). Actually, hereditary breast and ovarian cancer syndrome (HBOC) is a well-described hereditary cancer predisposition syndrome caused by mutations in *BRCA1 *and *BRCA2* genes.

The lifetime risk for women with *BRCA1* mutations is estimated to be about 72% for BC (95%CI, 65–79%) and 44% for OC (95%CI, 36–53%. The corresponding estimates for BRCA2 are 69% (95%CI,61–77%) and 17% (95%CI, 11–25%) respectively (9). A timely identification of BRCA-mutation carriers is therefore key in order to plan adequate risk-reduction strategies.

However, synchronous or metachronous BC and OC diagnoses have been documented also in the absence of a germline *BRCA* mutation, suggesting other common etiological factors such as hormonal and reproductive aspects and mutation of other genes involved in tumor suppression (10–12). We nowadays know several other genes whose germline mutations can increase the lifetime risk of breast and/or ovarian cancer, such as CHEK2, BRIP1, BARD1, ATM; RAD51C, RAD51D, PALB2 and the genes associated with familial hereditary non-polyposys colorectal cancer (MSH6, MSH2, and MLH1) (13). There are few studies describing cohorts of patients with synchronous or metachronous BC and OC. In the present paper, we describe clinico-pathological characteristics including *BRCA* status, treatments and clinical outcome of a multicentric cohort of patients diagnosed with synchronous or metachronous BC and OC.

## Materials and Methods

### Patients

We reviewed medical reports of patients with synchronous or metachronous BC and OC, diagnosed between 1981 and 2016 at two large Italian cancer centers: the Istituto Oncologico Veneto IRCCS (Padova) and the Istituto Europeo di Oncologia (Milano). Patients with borderline or non-epithelial OC and patients with *in situ* BC were excluded. According to the sequence of cancer diagnoses, patients were classified into three groups: *BC first* (BC followed by OC), *OC first* (OC followed by BC) and *synchronous* (time between the two diagnoses <4 months). When available, the *BRCA-*mutational status was also recorded.

Further information including tumor stage (according to the AJCC 7th Edition BC Staging and FIGO 2014 OC staging), histological type, tumor grade, hormonal/HER2 receptors status (BC), surgical and medical treatment were collected. Follow-up data including death cause were also gathered.

### Statistical Analysis

Statistical analysis was conducted using IBM SPSS Version 24. The association between variables was evaluated using the χ2 test or t test, as appropriate.

Overall survival (OS) from the first diagnosis was defined as the time interval from the first cancer diagnosis (BC or OC whichever first) to the date of death/last follow-up. We also evaluated the OS after the second diagnosis, calculated from the time of BC or OC diagnosis (whichever last) to death/last follow-up. For survival analyses, the hazard ratio (HR) and 95% confidence interval (95% CI) were calculated with the Cox regression model. Survival curves were estimated using the Kaplan–Meier model, and the log-rank test was used to test the differences between the groups. Level of significance was set at 0.05. The data cut-off for the survival events was May 2017.

## Results

### Patients Characteristics According to the Sequence of Breast Cancer and Ovarian Cancer Diagnoses

Two hundred and seventy patients were included and classified as follows according to the first cancer diagnosis: *BC first* n = 194 (72%), *OC first* n = 51 (19%), and *synchronous* n = 25 (9%). The clinico-pathological characteristics of the population according to BC/OC diagnosis sequence are summarized in **Table 1**.

**Table 1:** Clinico-pathological characteristics of patients according to BC/OC diagnosis sequence.

	Total n = 270	*BC First* n = 194	*OC First* n = 51	*Synchronous* n = 25	p	p^a^
	n (%)	n (%)	n (%)	n (%)	
**Age 1^st^ diagn.**					**<0.001**	
Mean (months) Range (months)	50 28–85	48 28–83	54 30–76	60 37–85		
**Age 2^nd^ diagn.**					**<0.001**	
Mean (months) Range (months)	58 36–85	57 38–84	61 36–79	60 37–85		
**OC Histology**					**0.002**	**<0.001**
Serous Endometrioid Indifferent. Other Total	188 (70.1%) 39 (14.6%) 16 (6.0 %) 25 (9.3%) 268	146 (75.3%) 18 (9.3%) 12 (6.2%) 18 (9.3%) 194	24 (49.0%) 16 (32.7%) 3 (6.1%) 6 (12.2%) 49	18 (72.0%) 5 (20.0%) 1 (4.0%) 1 (4.0%) 25		
**OC Stage**					**0.039**	**0.011**
**** I–II III–IV Total	81 (30.6%) 184 (69.4%) 265	52 (26.9%) 141 (73.1%) 193	22 (45.8%) 26 (45.8%) 48	7 (29.2%) 17 (70.8%) 24		
**OC Grade**					**<0.001**	**<0.001**
1–2 3 Total	46 (17.5%) 217 (82.5%) 263	22 (11.5%) 169 (88.5%) 191	20 (42.6%) 27 (57.4%) 47	4 (16.0%) 21 (84.0%) 25		
**BC Histology**					**0.038**	**0.017**
Ductal Lobular Other Total	212 (82.8%) 18 (7.0%) 26 (10.2%) 256	154 (85.1%) 9 (5.0%) 18 (9.9%) 181	35 (70.0%) 8 (16.0%) 7 (14.0%) 50	23 (92.0%) 1 (4.0%) 1 (4.0%) 25		
**BC Stage**					**0.022**	0.082
≤I ≥II Total	120 (51.7%) 112 (48.3%) 233	72 (45.9%) 85 (54.1%) 157	30 (60.0%) 20 (40.0%) 50	18 (72.0%) 7 (28.0%) 25		
**BC Grade**					0.204	0.087
1–2 3 Total	108 (48.0%) 117 (52.0) 225	70 (45.2%) 85 (54.8%) 155	29 (59.2) 20 (40.8) 49	9 (42.9%) 12 (57.1%) 21		
**BC Nodal status**					0.199	0.287
**** Negative Positive Total	162 (67.2%) 79 (32.3%) 241	106 (63.9%) 60 (36.1%) 166	36 (72.0%) 14 (28.0%) 50	20 (80.0%) 5 (20.0%) 25		
**BC HR**					0.051	0.054
Negative Positive Total	93 (37.5%) 155 (62.5%) 248	73 (42.4%) 99 (57.6%) 172	14 (27.5%) 37 (72.5%) 51	6 (24.0%) 19 (76.0%) 25		
**BC Her2**					0.390	0.462
**** Negative Positive Total	164 (89.6%) 19 (10.4%) 183	103 (89.6%) 12 (10.4%) 115	42(93.3%) 3 (6.7%) 45	19 (82.6%) 4 (17.4%) 23		
**TNBC**					0.076	0.076
**** No TN TN Total	163 (67.6%) 78 (32.4%) 241	104 (63%) 61 (37%) 165	39 (76.5%) 12 (23.5%) 51	20 (80.0%) 5 (20.0%) 25		
**BRCA status**					**0.006**	**0.003**
Wild Type Mutated **** Total	70 (38.5%) 112 (61.5%) 182	42 (31.6%) 91 (68.4%) 133	19 (59.4%) 13 (40.6%) 32	9 (52.9%) 8 (47.1%) 17		
**BRCA mutation**					0.066	0.804
**** BRCA1 BRCA2 BRCA1&2 Unknown Total	72 (64.3%) 31 (27.8%) 3 (2.7%) 6 (5.4%) 112	60 (65.9%) 23 (25.3%) 2 (2.2%) 6 (6.6%) 91	10 (76.9%) 3 (23.1) 0 0 13	2 (25%) 5 (62.5%) 1 (12.5%) 0 8		

n, number; OC, ovarian cancer; BC, breast cancer; TNBC, triple negative breast cancer; HR, hormone receptors.

a Excluding synchronous group (BC first vs OC first).

*BC first* patients presented the youngest age at first and second diagnosis compared to the other groups (p < 0.001 in both cases).

The *BC first* and *synchronous* groups, as compared to *OC first* group, showed a significantly higher frequency of OC of serous histology (75.3 and 72.0 *vs* 49%, p = 0.002), high grade (88.5 and 84.0 *vs* 57.4%, p < 0.001) and FIGO stage ≥III (73.1 and 70.8 *vs* 45.8%, p = 0.039).

The most frequent histotype of BC was ductal infiltrating carcinoma in all groups; however, in the *OC first* group, lobular histotype was more represented (16.0 *vs* 5.0% in *BC first* and 4.0% in *synchronous*; p = 0.038). More than a half (54.1%) of *BC first* patients presented stage >II BC at diagnosis as compared to 40.0 and 28.0% of patients in the *OC first* and *synchronous* groups, respectively (p = 0.022). *BC first* patients showed the highest proportion of TNBC (37 *vs* 23.5% and 20.0% in the *OC first* and *synchronous* groups, p = 0.076).

### Treatments According to the Sequence of Breast Cancer and Ovarian Cancer Diagnoses

The majority of patients received a platinum-based chemotherapy for OC (92.4%). More patients in the *OC first* group were treated with other chemotherapy regimens (8.2%), reflecting the different histological patterns.

As expected, since treatment selection for BC is based on tumor phenotype, BC treatment was significantly different among the cancer sequence groups. Indeed, more than 70% of *BC first* and *synchronous* patients were treated with adjuvant chemotherapy reflecting the higher prevalence of TNBC subtype in this group. **Table 2** shows data regarding medical and surgical treatments.

**Table 2:** Medical and surgical treatments according to BC/OC diagnosis sequence.

	Total n = 270	*BC First* n = 194	*OC First* n = 51	*Synchronous* n = 25		
	**n (%)**	**n (%)**	**n (%)**	**N (%)**	**p**	**p** ^a^
**OC 1^st^ Line CT**					**0.010**	**0.003**
Plat-based Other None Total	231 (92.4%) 5 (2.0%) 14 (5.6%) 250	166 (93.3%) 1 (0.6%) 11 (6.2%) 178	44 (89.9%) 4 (8.2%) 1 (2.0%) 49	21 (91.3%) 0 (0.0%) 2 (8.7%) 23		
**BC Surgery**					0.212	0.098
Mastectomy Conservative None Total	70 (26.8%) 190 (72.8%) 1 (0.4%) 261	54 (29.2%) 131 (70.8%) 0 185	11 (21.6%) 39 (76.5%) 1 (2.0%) 51	5 (20.0%) 20 (80.0%) 0 (0.0%) 25		
**HT for BC**					**0.004**	**0.007**
Yes No Total	135 (55.6%) 108 (44.4%) 243	82 (48.5%) 87 (51.5%) 169	35 (70.0%) 15 (30.0%) 50	18 (75.0%) 6 (25.0%) 24		
**CT for BC**					**0.004**	**0.002**
Yes No Total	163 (67.4%) 79 (32.6%) 242	121 (72.0%) 47 (28.0%) 168	24 (48.0%) 26 (52.0%) 50	18 (75.0%) 6 (25.0%) 24		

n, number; OC, ovarian cancer; CT, chemotherapy; BC, breast cancer; HT, hormonal therapy.

a Excluding synchronous group (BC first vs OC first).

### Patients With Known BRCA Status: Baseline Characteristics and Treatment

For n = 182 (67.4%) patients, the status of *BRCA1/2* genes was available. As shown in **Table 1**, n = 112 patients (61.5%) were *BRCA* mutated (64.3% *BRCA1*, 27.8% *BRCA2* and 2.7% *BRCA 1&2*) and n = 70 (38.5%) were wild type. The frequency of *BRCA* mutation carriers was significantly different in the three groups: 68.4% of *BC first* patients, 40.6% of *OC first* patients and 47.1% of patients in the *synchronous* group (p = 0.006). For the vast majority of patients with available *BRCA* status, the genetic test was performed after both tumors had been diagnosed (85.2%), with no difference between the groups (p = 0.312).

The evaluation of clinico-pathological characteristics based on the mutational status of *BRCA1/2* genes is reported in **Table 3**.

**Table 3:** Clinico-pathological characteristics of patients according to BRCA mutational status.

	Total n = 182	BRCA *Wild Type* n = 70	BRCA *Mutated* n = 112	P
	n (%)	n (%)	n (%)
**Age 1^st^ diagn.**				**<0.001**
Mean (months) Range (months)	49 28–85	54 34–85	47 28–80	
**Age 2^nd^ diagn.**				**<0.001**
Mean (months) Range (months)	58 36–85	62 42–85	55 36–81	
**OC Histolgy**				**0.006**
Serous Endometrioid Indifferent. Other Total	112 (68.3%) 23 (14.0%) 11 (11.0%) 18 (6.7%) 164	33 (55.0%) 12 (20.0%) 3 (5.0%) 12 (20.0%) 60	79 (76.0%) 11 (10.6%) 8 (7.7%) 6 (5.8%) 104	
**OC Stage**				**0.002**
I–II III–IV Total	53 (32.7%) 109 (67.3%) 162	28 (47.5%) 31 (52.5%) 59	25 (24.3%) 78 (75.7%) 103	
**OC Grade**				**<0.001**
1–2 3 Total	31 (19.1%) 131 (80.9%) 162	23 (39.0%) 36 (61.0%) 59	8 (7.8%) 95 (92.2%) 103	
**BC Histology**				0.421
Ductal Lobular Other Total	127 (83.0%) 12 (7.8%) 14 (9.2%) 153	46 (78.0%) 6 (10.2%) 7 (11.9%) 59	81 (86.2%) 6 (6.4%) 7 (7.4%) 94	
**BC Stage**				0.504
I ≤ ≥ II Total	64 (48.5%) 68 (51.5%) 132	20 (44.4%) 25 (55.6%) 45	44 (50.6%) 43 (49.4%) 87	
**BC Grade**				**<0.001**
1–2 3 Total	66 (48.9%) 69 (51.1%) 135	35 (70.0%) 15 (30.0%) 50	31 (36.5%) 54 (63.5%) 85	
**BC Lymph.**				0.642
Negative Positive Total	90 (65.7%) 47 (34.3%) 137	29 (66.0%) 17 (37.0%) 46	61 (67.0%) 30 (33.0%) 91	
**BC HR**				**<0.001**
Negative Positive Total	54 (37.2%) 91 (62.8%) 145	9 (16.7%) 45 (83.3%) 54	45 (49.5%) 46 (50.5%) 91	
**BC Her2**				0.345
Negative Positive Total	86 (89.6%) 10 (10.4%) 96	30 (93.8%) 2 (6.3%) 32	56 (87.5%) 8 (12.5%) 64	
**TNBC**				**0.001**
No TN TN Total	96 (69.1%) 43 (30.9%) 139	45 (86.5%) 7 (13.5%) 52	51 (58.6%) 36 (41.4%) 87	

number n, ovarian cancer OC, breast cancer BC, triple negative breast cancer TNBC, hormonal receptors HR.

The mean age at the first and second diagnoses was lower in the *BRCA* mutated group (p < 0.001).

*BRCA* mutated patients had more frequent OC of advanced stage (FIGO stage ≥III 75.7 *vs* 52.5%; p = 0.002), serous histology (76.0 *vs* 55.0%, p = 0.006) and high grade (92.2 *vs* 61.0%, p < 0.001).

Regarding BC, as expected, *BRCA* mutated patients presented more TN (41.4 *vs* 13.5%; p = 0.001) and grade 3 (63.5 *vs* 30.0%; p < 0.001) tumors, reflecting the higher prevalence of *BRCA1* mutation (BRCA1 64.3%). **Table 4** shows medical and surgical treatments according to *BRCA* status. No difference was observed in terms of chemotherapy for OC, with the majority of patients being treated with 1^st^ line platinum-based CT (92%). The majority of *BRCA* wild type patients received HT for BC. CT use was more frequent in *BRCA* mutated patients (75%) reflecting the higher prevalence of TNBC subtype in this group. Surgical treatment for BC consisted in conservative surgery in most cases (66.4% of *BRCA* mutated and 66.7% of *BRCA* wild type), and this finding is consistent with the fact that BRCA status was unknown at time of surgery for most of the patients.

**Table 4:** Medical and surgical treatments according to BRCA mutational status.

	Total N = 182	BRCA *Wild Type* N = 112	BRCA *Mutated* N = 70	p
	n (%)	n (%)	n (%)
**OC 1^st^ Line CT**				0.156
Platinum Based Other None Total	150 (92.0%) 5 (3.1%) 8 (4.9%) 163	52 (86.7%) 3 (5.0%) 5 (8.3%) 60	98 (95.1%) 2 (1.9%) 3 (2.9%) 103	
**BC Surgery**				0.435
Mastectomy Conservative None Total	57 (32.9%) 115 (66.5%) 1 (0.6%) 173	21 (31.8%) 44 (66.7%) 1 (1.5%) 66	36 (33.6%) 71 (66.4%) 0 (0.0%) 107	
**HT for BC**				**0.001**
Yes No Total	87 (54.4%) 73 (45.6%) 160	42 (71.2%) 17 (28.8%) 59	45 (44.6%) 56 (55.4%) 101	
**CT for BC**				**0.007**
Yes No Total	107 (67.3%) 52 (32.7%) 159	32 (54.2%) 27 (45.8%) 59	75 (75.0%) 25 (25.0%) 100	

n, number; OC, ovarian cancer; CT, chemotherapy; BC, breast cancer; HT, hormonal therapy.

## Interval Between Cancer Diagnoses

**Figure 1:**
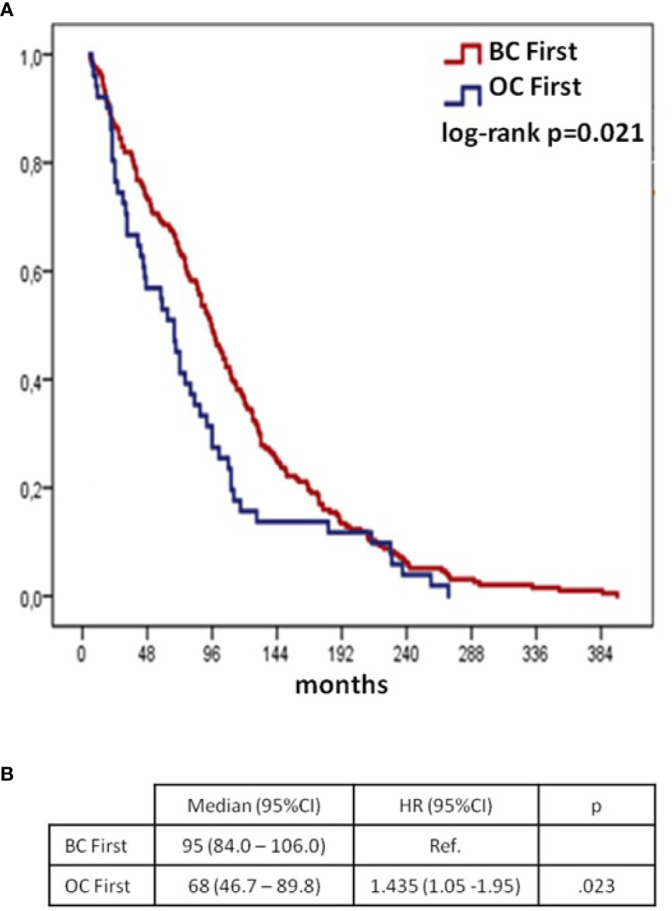
Time from 1^st^ to 2^nd^ cancer diagnoses according to BC/OC diagnosis sequence (*synchronous* group excluded): Kaplan–Meier curves **(A)** and Cox regression model **(B)** BC, breast cancer; OC, ovarian cancer; CI, confidential interval; H, hazard ratio; OS, overall survival.

Median time interval from first to second diagnosis in overall cohort (including *synchronou*s patients) was 78 months (95%CI 67.6–88.4). When comparing *BC first* group to the *OC first* group, the time interval was longer in *BC first*: median 95 months (95%CI 84.0–106.0 months) *vs* 68 months (95%CI 46.7–89.8), respectively (**Figure 1**, p = 0.021).

**Figure 2:**
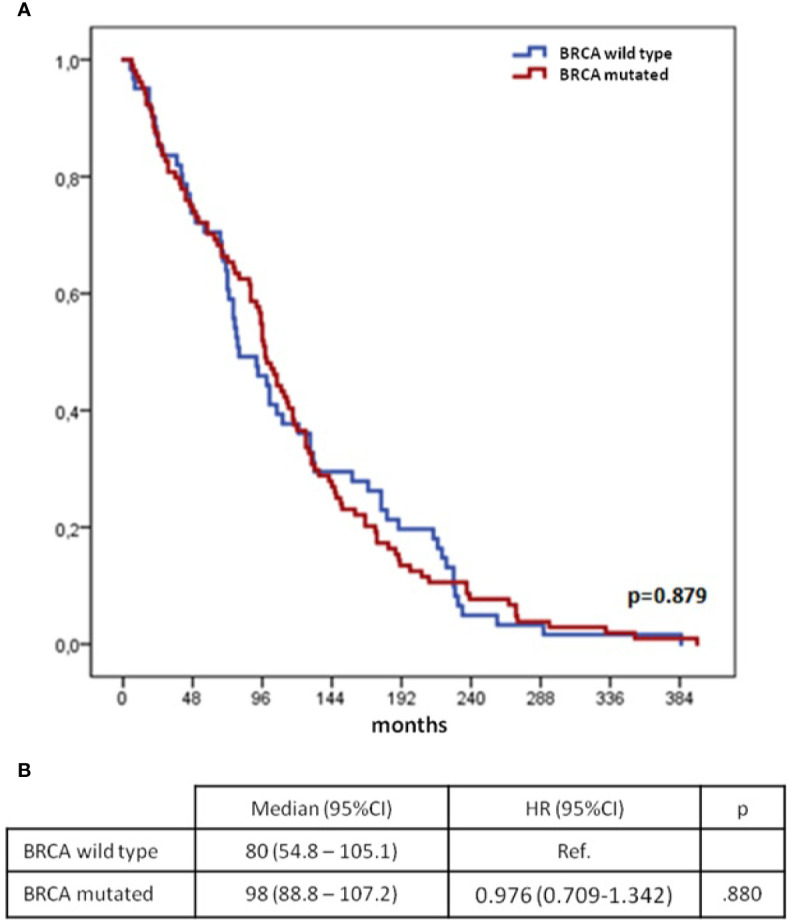
Time from 1^st^ to 2^nd^ cancer diagnoses according to mutational status of BRCA genes (*synchronous* group excluded): Kaplan–Meier curves **(A)** and Cox regression model **(B)** BC, breast cancer; OC, ovarian cancer; CI, confidential interval; H, hazard ratio; OS, overall survival.

Median time between diagnoses was 96 months (95%CI 85.6–106.4) for the n=182 patients with available *BRCA* gene status and was similar in *BRCA* mutated and wild type patients (**Figure 2**).

### Overall Survival Analysis

The median duration of follow-up in the entire cohort of patients was 16 years (95%CI 14.8–17.2) from the first diagnosis and 7.1 years (95%CI 5.6–8.5) from the second; for the *BC first* group, 16.3 years (95%CI 14.8–17.8) and 6.4 years (95%CI 4.8–8.1); for the *OC first* group, 16.3 years (95%CI 10.4–22.1) and 8.5 years (95%CI 5.9–11.1); for *synchronous* group 9.6 years (95%CI 4.6–14.5) and 9.6 years (95%CI 5.1–14.1). At the cut-off date, 105 patients (39.2%) had died. Patients more frequently died from OC-related consequences in all groups (**Table 5**).

**Table 5:** Cause of deaths according to BC/OC diagnosis sequence.

Death Cause	Total deaths n = 105	*BC First* n = 77	*OC First* n = 16	*Synchronous* n = 12	p
	n (%)	n (%)	n (%)	n (%)
OC	74 (70.5%)	56 (72.7%)	9 (56.3%)	9 (75.0%)	0.163
BC	8 (7.6%)	5 (6.5%)	3 (18.8%)	0 (0.0%)	
No cancer related	9 (8.6%)	4 (5.2%)	3 (18.8%)	2 (8.3%)	
Unknown	14 (13.3%)	12 (15.6%)	1 (6.3%)	1 (8.3%)	

n, number; OC, ovarian cancer; BC, breast cancer.

**Figure 3:**
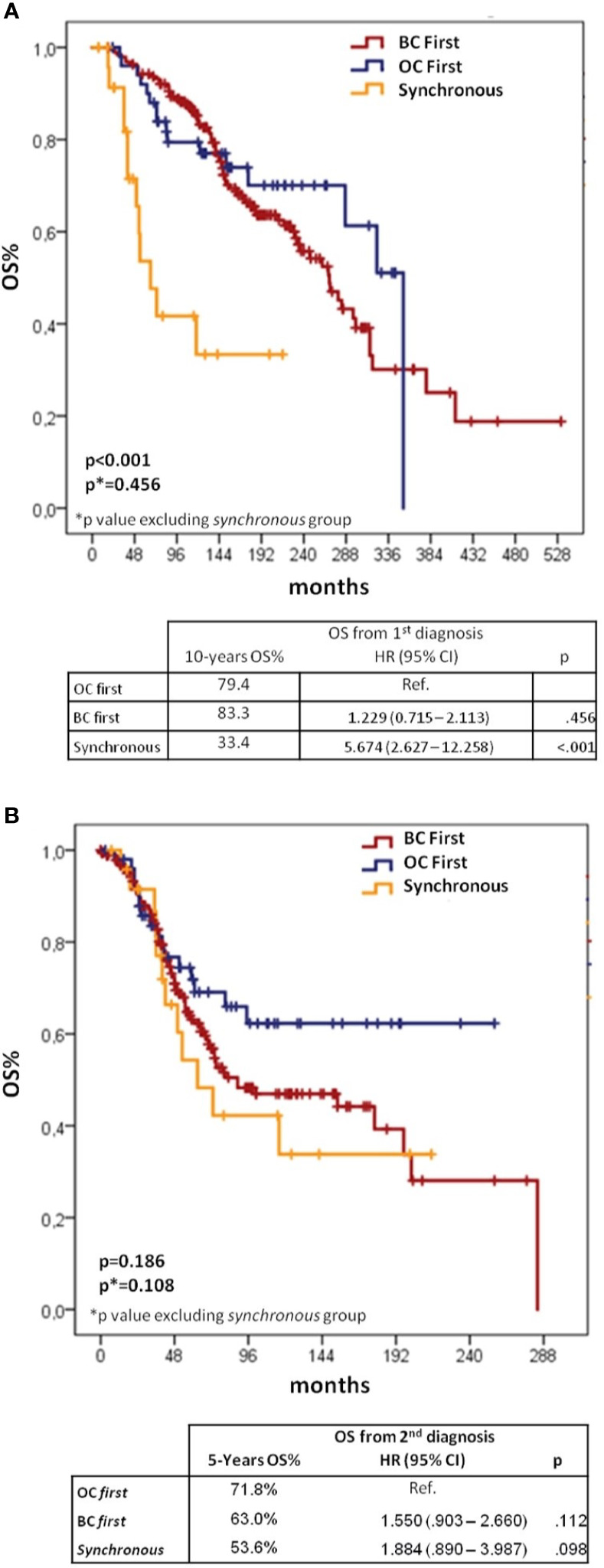
Kaplan–Meier and Cox regression for OS from 1^st^ **(A)** and 2^nd^ diagnoses **(B)** according to BC/OC diagnosis sequence. BC, breast cancer; OC, ovarian cancer; CI, confidential interval; H, hazard ratio; OS, overall survival.

Kaplan–Meier curves and Cox models for OS analyses from first and second diagnoses according to the sequence of cancer diagnoses are shown in **Figure 3**.

When considering OS from first cancer diagnosis, 10-yr survival rates were 83.3% for *BC first*, 79.4% for *OC first* and 33.4% for *synchronous* groups (log-rank p < 0.001 overall, p = 0.456 for the comparison between *BC first* and *OC first*). The Cox-regression model showed significantly worse OS for *synchronous vs OC first* group (HR 5.674, 95%CI 2.627–12.258, p < 0.001), but no difference between *BC first* and *OC first* groups (HR 1.229, 95%CI 0.715–2.113, p = 0.456).

When assessing OS from the second diagnosis, the *OC first* group showed the best outcome with a 5-yr survival rate of 71.8%, followed by the *BC first* group (63.0%) and the *synchronous* group (53.6%), although differences between groups were not statistically significant.

### Prognostic Factors for Overall Survival

**Figure 4:**
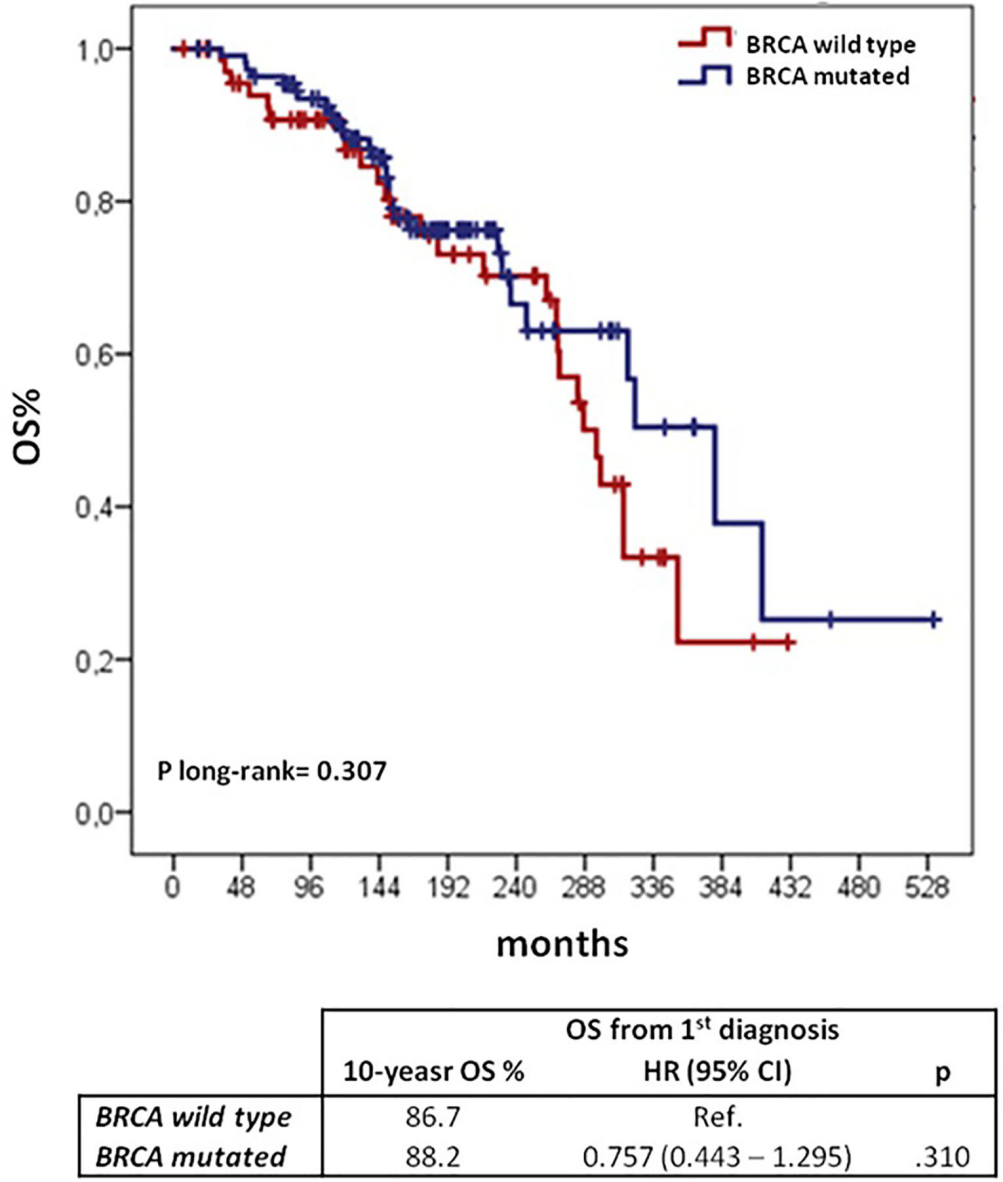
OS from the 1^st^ cancer diagnosis according to BRCA mutational status: Kaplan–Meier curves and Cox regression model. BC, breast cancer; OC, ovarian cancer; CI, confidential interval; H, hazard ratio; OS, overall survival.

We investigated other potential prognostic factors for OS after first cancer onset, **Table 6** shows univariate and multivariate analyses. Older age (>50 years) at first diagnosis and advanced OC (FIGO stage ≥III) proved to be independent prognostic factors of poorer survival. Comparing *BRCA* mutation carriers and wild type patients we found no difference in OS from the first diagnosis (10-yr OS of 88.2 and 86.7%, respectively; log-rank p = 0.307) (**Figure 4**).

**Table 6:** Univariate and multivariate analysis for OS from 1st cancer diagnosis.

	Univariate Analysis			Multivariate Analysis		
	HR	95% CI	p	HR	95% CI	P
**Age at 1^st^ diagnosis**						
**50≤ *vs* >50 years**	1.817	1.235–2.674	**0.002**	1.843	1.251–2.714	**0.002**
**OC Stage**						
**<III *vs* ≥III**	2.991	1.720–5.203	**<0.001**	2.687	1.615–4.469	<0**.001**
**OC Histology**						
**Serous/Indiff *vs* Other**	1.597	0.979–2.606	0.061			
**OC Grade**						
**1–2 *vs* 3**	1.247	0.723–2.152	0.428			
**BC Stage**						
**I *vs* ≥II**	0.996	0.640–1.550	0.958			
**BC Grade**						
**1–2 *vs* 3**	1.028	0.644–1.640	0.908			
**BC Hormone Receptors Status**						
**Neg. *vs* Pos.**	0.992	0.635–1.552	0.973			
**BC Ki67**						
**≤14 *vs* >14%**	0.950	0.511–1.768	0.872			

OC, ovarian cancer; BC, breast cancer; HR, hazard ratio; CI, confidence interval.

## Discussion

There is limited information in the literature regarding clinical presentation and outcome of patients with synchronous or metachronous OC and BC. This is the second largest cohort, following that reported by Liou et al. in 2006. The majority of our cohort was represented by patients diagnosed with BC followed by OC (72%), consistently with other data (14). Up to 68.5% of patients with available information on BRCA status in this group harbored a *BRCA* mutation, as compared to 41% of OC first and 47% of synchronous patients. The different prevalence of BRCA mutated patients, especially BRCA1 mutated, may account for some of the differences observed in clinico-pathological characteristics between the groups, with *BC first* patients showing younger age at 1^st^ and 2^nd^ diagnoses, more aggressive OC features and a higher prevalence of TNBC.

A previous study also described aggressive OC features in patients diagnosed with BC followed by OC (14).

The interval between the two diagnoses was significantly longer in the *BC first* group as compared to the *OC first* group. These data are in contrast with previous findings. Olawaiye A et al. found a significantly longer time interval in women who developed OC before BC (7 *vs* 4 years), but the sample size was limited (49 patients only) (15). To the other hand, Liou et al. did not show any difference in the time from first to second diagnoses (14).

In our cohort, *BC first* patients presented younger age at both first and second diagnosis as compared to other groups; however, the magnitude of diagnosis anticipation, appeared larger for BC diagnosis than for OC cancer diagnosis, possibly justifying the observation of a longer time to second diagnosis. Indeed, it is well recognized that *BRCA* carriers, who were more represented in the *BC first* group, may experience BC at very young age (16). Despite the longer time to second diagnosis in *BC first* group, we did not detect any difference in OS from first diagnosis between *BC first* and *OC first* groups. In the work by Liou et al., where no difference in interval between the two diagnoses was observed, *BC first* group had a poorer survival from first diagnosis than women in *OC first* group (14). Data from our work and the one by Lious et al.’s are consistent with the assumption that the subsequent OC diagnosis in *BC first* group is the main determinant of OS. Indeed, patients in this group had poor prognostic clinico-pathological OC characteristics and OC-related deaths, although being the most frequent death cause in the whole population, accounted for 72.7% of the OS events in *BC first* patients as compared to 56.3% of the OS events in the *OC first* group. Therefore, in the *BC first* group, the potential favorable effect of a long time interval between the two diagnoses was somehow neutralized by the poor prognosis that these patients experienced after OC diagnosis.

Several studies have reported more favorable survival outcomes among *BRCA* mutated OC patients compared with patients affected by sporadic OC (17–19). Interestingly, Zaaijer LH et al. observed a worse outcome for *BRCA* carriers *vs BRCA* non-carriers among patients diagnosed with OC after BC (20). In our paper, we did not find any difference in OS between *BRCA* mutated and wild type patients with both breast and ovarian cancer. Our data together with those of Zaaijer LH et al., suggest that the evidence of a better prognosis of *BRCA* mutated patients with OC might not be confirmed in cases with a metachronous BC. Of course, our cohort goes back to a pre-PARPi era and we can assume that because of their high activity in BRCA mutated OC patients they could overwhelm this unfavorable prognostic factor. On the other hand, it will be interesting to observe if after the introduction of PARPi for OC treatment, there will be an increase of BC metachronous diagnoses because of the prolonged survival or if, on the contrary, they could have a sort of “chemo-preventive” effect, reducing the incidence of breast cancer after ovarian cancer.

As already discussed, in our series survival is dominated by OC related mortality. These findings can be useful for adequately counsel *BRCA* mutated patients with a first diagnosis of breast cancer or ovarian cancer on how to balance potential benefits and harms of subsequent preventive measures. Appropriate surveillance and prophylactic oophorectomy are recommended for BC survivors with *BRCA* mutation. In case of a first diagnosis of BC in patient with a *BRCA* mutation who has not yet undergone prophylactic oophorectomy, our data strongly support to recommend this procedure, since subsequent OC was the main determinant of overall survival. This is particularly relevant if we consider that survival of breast cancer patients is constantly improving over time thanks to surveillance and advances in systemic therapies (20). With regard to the optimal timing of prophylactic oophorectomy after a BC diagnosis in a *BRCA* mutated patient, our data support the same timing as in healthy *BRCA* carriers. Indeed, the age at OC diagnosis in patients with a previous BC in our study is consistent to the age of ovarian cancer diagnosis in *BRCA* mutated carriers. On the other hand, counseling in patients with *BRCA-*associated OC is more complex, because it should address not only the subsequent risk of BC but also the consideration of this risk against the OC prognosis. In our study the rate of *BRCA* mutated patients was the lowest in the *OC first* group; this observation is consistent with the results of previous studies, showing that metachronous BC in *BRCA* carriers with previous OC is infrequent, occurring in around 10% of the patients. Moreover, the same studies also confirmed that survival of this patients is dominated by OC (21, 22). McGee J et al. recently showed that in *BRCA* mutation carrying patients diagnosed with stage III/IV OC, the chance of dying for all causes was reduced by less than 1% with breast MRI and by less than 2% with mastectomy. The benefits of more aggressive preventive measures, as prophylactic bilateral mastectomy or intensive radiological surveillance, are expected to be small in terms of lives saved in particular in presence of poor prognostic factors like age >50 years and OC ≥III FIGO stage (23). An adequate counseling should account for these aspects.

Finally, the importance of offering genetic test to patients at risk of *BRCA1/2* mutation should be underlined. Since the introduction of the *BRCA* genetic test until recently, the main criteria to allow access to the test was based on family history. This in part explains why in our cohort of patients *BRCA* status was not available for all patients, and when performed, genetic test occurred most frequently after the second cancer diagnosis. More recently, genetic test eligibility criteria have been expanded. With regard to OC patients, the observation that about 12% of patients with high-grade serous OC were mutated unless there was a family history (24, 25) led to recommend the test for all patients with this diagnosis, irrespectively of familial history. With regard to BC, newly introduced criteria include patients with TNBC diagnosed at the age of 60 or younger, irrespectively of familial history (26, 27).

In conclusion, our study reports data from a large cohort of patients with synchronous and metachronous BC and OC diagnosed in a time span covering recent years. Our data may be useful in order to plan and carry out adequate and timely surveillance programs and preventive measures.

## Data Availability Statement

The raw data supporting the conclusions of this article will be made available by the authors, without undue reservation.

## Author Contributions

GT acquired the data, analyzed and interpreted the data, and wrote the original draft. MD developed the methodology, analyzed and interpreted the data, and reviewed the manuscript. ZB, GF, MM, MN, and FP provided the resources. VG conceptualized and designed the study, developed the methodology, reviewed the manuscript, and supervised the study. NC supervised the study and provided the resources. All authors contributed to the article and approved the submitted version.

## Conflict of Interest 

GT has received fees from AstraZeneca, Tesaro and GSK for consultancy role and participation on advisory boards. MD has received fees from EliLilly for consultancy role and participation on advisory boards, fees from Genomic Health for consultancy role, fees from Celgene for participation on advisory. FP has received remuneration from Ipsen and fees from Roche and AstraZeneca for consultancy role and participation on advisory boards. VG has received fees from EliLilly, Roche, and Novartis.

The remaining authors declare that the research was conducted in the absence of any commercial or financial relationships that could be construed as a potential conflict of interest.
